# Functional analysis of seven regulators of G protein signaling (RGSs) in the nematode-trapping fungus *Arthrobotrys oligospora*

**DOI:** 10.1080/21505594.2021.1948667

**Published:** 2021-07-05

**Authors:** Ni Ma, Yining Zhao, Yunchuan Wang, Le Yang, Dongni Li, Jiangliu Yang, Kexin Jiang, Ke-Qin Zhang, Jinkui Yang

**Affiliations:** a State Key Laboratory for Conservation and Utilization of Bio-Resources, and Key Laboratory for Microbial Resources of the Ministry of Education, Yunnan University, Kunming P. R. China; b School of Life Sciences, Yunnan University, Kunming P. R. China; c Institute of Medical Biology, Chinese Academy of Medical Sciences & Peking Union Medical College, Kunming P. R. China

**Keywords:** *Arthrobotrys oligospora*, regulator of g protein signaling (rgs), conidiation, trap formation, transcriptional analysis, *gas1* family genes

## Abstract

Regulators of G protein signaling (RGSs) are proteins that negatively regulate G protein signal transduction. In this study, seven putative RGSs were characterized in the nematode-trapping (NT) fungus, *Arthrobotrys oligospora*. Deleting *Rgs* genes significantly increased intracellular cAMP levels, and caused defects in mycelia growth, stress resistance, conidiation, trap formation, and nematocidal activity. In particular, the Δ*AoFlbA* mutant was unable to produce conidia and traps. Transcriptomic analysis showed that amino acid metabolic and biosynthetic processes were significantly enriched in the Δ*AoFlbA* mutant compared to WT. Interestingly, *Gas1* family genes are significantly expanded in *A. oligospora* and other NT fungi that produce adhesive traps, and are differentially expressed during trap formation in *A. oligospora*. Disruption of two *Gas1* genes resulted in defective conidiation, trap formation, and pathogenicity. Our results indicate that RGSs play pleiotropic roles in regulating *A. oligospora* mycelial growth, development, and pathogenicity. Further, AoFlbA is a prominent member and required for conidiation and trap formation, possibly by regulating amino acid metabolism and biosynthesis. Our results provide a basis for elucidating the signaling mechanism of vegetative growth, lifestyle transition, and pathogenicity in NT fungi.

## Introduction

Fungi can sense changes in various physical and chemical stimuli in the environment, which regulate downstream gene expression in the cell and ultimately regulate their growth and development in response to environmental changes [1]. This biological process involves multiple signal transduction pathways. Among them, heterotrimeric G protein (G protein) signaling is the most conserved signal transduction mechanism in fungi [2,3]. G proteins are composed of three subunits, α (Gα), β (Gβ), and γ (Gγ), which exist in a complex in the inactive state [1]. G-protein-coupled receptors (GPCRs) are the largest class of cell surface receptors in eukaryotes. They can sense environmental cues and initiate intracellular G protein signaling to coordinate a biological response [3]. In the presence of a ligand, GPCRs can accelerate the exchange of GTP for GDP on the Gα protein and dissociation of the Gα and Gβγ dimer. Both the Gα-GTP and Gβγ heterocomplexes can transmit signals to downstream effectors, including cAMP-dependent protein kinase A (PKA), ion channels, and mitogen-activated protein kinases [1,3]. In fungi, G proteins are associated with numerous biological processes, including sexual and asexual reproduction, virulence, and response to external signal stimuli [1,4,5]. For example, GpaB (Gα) regulates growth and asexual development via the cAMP/PKA signal transduction pathway in *Aspergillus fumigatus* [6], whereas SfaD (Gβ) and GpgA (Gγ) regulate trehalose hydrolysis, conidial germination, hyphal growth, and gliotoxin biosynthesis [7]. G protein signaling pathways are transiently regulated by regulators of G-protein signaling (RGSs). This is a large family of proteins with a conserved RGS structural domain, which plays a negative role in G protein signaling [8,9] due to its GTPase activator (GPA) activity. The GPA activity accelerates GTP hydrolysis at the Gα subunit, thereby limiting the intensity and duration of G protein activation and inactivating or terminating downstream signals [10,11]. GPCRs, G proteins, and RGSs are the key components of G protein signaling and highly conserved in most filamentous fungi [1,12].

Since the first RGS was found in *Saccharomyces cerevisiae* [13], numerous proteins with similar structure and function to RGS have been identified in yeast and filamentous fungi [12,14]. Four RGSs, Sst2, Rgs2, Rax1, and Mdm1, were identified in *S. cerevisiae*, and Sst2 serves as the principal regulator of mating pheromone signaling [2,13]. Similarly, five RGSs (RgsA, FlbA, RgsB, RgsC, and GprK) were characterized in *Aspergillus nidulans*, and they play important roles in vegetative growth, sexual and asexual development, and pigment production [15,16]. Since then, the roles of RGSs have been characterized in several pathogenic fungi. For example, eight RGSs were identified in *Magnaporthe oryzae* (syn. *M. grisea*), where they play roles in pathogenicity, spore formation and germination, sexual reproduction, and secondary metabolite synthesis. Among them, MoRgs1, MoRgs3, MoRgs4, and MoRgs7 play a key role in the pathogenicity of this fungus [17]. Seven RGSs were identified in *Gibberella zeae* (ana. *Fusarium graminearum*); they are involved in the regulation of vegetative growth, spore morphogenesis, pathogenicity, and toxin synthesis to varying degrees [14]. Likewise, six RGSs involved in the regulation of fungal growth, asexual development, germination, stress tolerance, and virulence have been identified in *A. fumigatus* [12]. Among these RGSs, FgFlbA is the most important member and has a significant effect on growth, conidiation, toxin synthesis, and virulence [14,17]. These studies show that RGSs are involved in diverse biological processes, including vegetative growth, sporulation, secondary metabolite synthesis, and pathogenicity.

Nematode-trapping (NT) fungi are a heterogeneous group of organisms that are broadly distributed in terrestrial and aquatic ecosystems [18]. These fungi can develop specific trapping devices (traps) from vegetative mycelia, such as adhesive networks, adhesive knobs, and constricting rings, for nematode predation [19,20]. Recently, enrichment analysis showed that genes encoding adhesions and Gas1 proteins are highly enriched in several NT fungi that produce adhesive traps, such as *Arthrobotrys oligospora, Dactylellina entomopaga*, and *Dactylellina cionopaga* [21]. *A. oligospora* is a typical NT fungi species, which captures nematodes by producing adhesive networks [18]. In our previous study, proteomic analysis of *A. oligospora* showed that multiple signal transduction pathways are involved in the regulation of trap formation [22]. Recently, several signaling proteins associated with G protein signaling, such as mitogen-activated protein kinases Slt2 [23], Hog1 [24], and Ime2 [25], calcium/calmodulin-dependent protein kinases [26], and Gβ [20], were proven to regulate trap formation and pathogenicity in *A. oligospora*. However, the functions of RGSs in *A. oligospora* and other NT Fungi are unknown.

In this study, seven putative RGSs were retrieved from the *A. oligospora* genome, and their roles were identified by gene knockout and multi-phenotypic analyses. The gene-deleted mutants (Δ*AoRgs*) showed significant differences from the wild-type (WT) strain in mycelial growth, conidiation, stress response, and pathogenicity. In particular, the Δ*AoFlbA* mutant lost the ability to produce traps for nematode predation. To probe the regulatory mechanism of AoFlbA in *A. oligospora*, we compared the transcriptomic profile of the WT and Δ*AoFlbA* mutant strain by RNA-sequencing. Moreover, *Gas1* family genes are differentially expressed during *A. oligospora* trap formation. Deleting two *Gas1* genes resulted in significantly reduced conidiation, trap formation, and pathogenicity. Our analysis showed that these seven RGSs play important roles in vegetative growth, development, and differentiation in *A. oligospora*.

## Materials and methods

### Fungal strains, media, and culture conditions

*Arthrobotrys oligospora* (ATCC24927) and its derived mutants were maintained in PDA, TG (1% tryptone and 1% glucose), and TYGA (10 g/L tryptone, 5 g/L yeast extract, 10 g/L glucose, 5 g/L molasses, and 20 g/L agar) media at 28°C. Uracil-deficient *S. cerevisiae* FY834 was used to construct the knockout vector [27] and was cultured in YPD (10 g/L yeast extract, 20 g/L peptone, and 20 g/L dextrose). *Escherichia coli* strain DH5⍺ was used to generate the pCSN44 plasmid, which contains the hygromycin resistance gene (*hph*) and the plasmid PRS426 used to construct the knockout vector. FY834 colonies containing the correct knockout vector were screened using SC-Ura plates, as previously described [27]. Liquid TG medium was used to prepare the mycelium for protoplast production or DNA extraction. PADS medium (PDA supplemented with 0.6 M sucrose and 5 g/L molasses) was supplemented with 200 μg/mL hygromycin for protoplast regeneration and positive transformant screening. Oatmeal water medium was used to culture the nematode *Caenorhabditis elegans*, which was used for trap induction and bioassays.

### Sequence and cluster analysis of RGSs in A. oligospora

Using orthologous RGS and RGS-like proteins from model fungi, such as *A. nidulans, M. oryzae*, and *S. cerevisiae*, as queries, the putative RGSs were retrieved from *A. oligospora*. The theoretical isoelectric point and MW of RGSs in *A. oligospora* were calculated using the online pI/MW tool (http://www.expasy.ch/tools/pi_tool.html). The conserved domains of RGSs were predicted using InterProScan (http://www.ebi.ac.uk/interpro/). The orthologous RGSs from different fungi were blasted and downloaded from GenBank, and a neighbor-joining tree was constructed using Mega v7.0 [28].

### Vector construction and targeted gene deletion

The seven deletion vectors were constructed as described previously [27]. Specifically, the upstream and downstream regions of the target gene and the hygromycin resistance gene cassette (*hph*) were amplified by PCR using the primer pairs shown in Table S2. The PRS426 plasmid was linearized using the restriction enzymes *Eco*RI and *Xho*I. The homologous target gene fragments, linearized PRS426 plasmid, and the *hph* fragment were transformed into *S. cerevisiae* competent cells by electroporation. The correct plasmid was screened on SC-Ura plates and transformed into *E. coli* DH5⍺ for storage. The final deletion cassette was amplified by PCR using 5 F/3 R primer pairs (Table S2) and was transformed into *A. oligospora* protoplasts as previously described [29]. Hygromycin-resistant transformants were screened in PDAS plates and further confirmed by PCR amplification using the verification primers YZF/YZR (Table S2) and Southern blot analysis, as described previously [30,31].

### Comparison of mycelial growth, colony morphology, and conidia yield

Fungal strains were incubated on PDA, TYGA, and TG plates at 28°C, and the colony diameter was determined at 24 h intervals. To evaluate the sporulation ability of fungal strains, fungal blocks of the same size were individually inoculated on CMY medium (20 g/L maizena, 20 g/L agar, and 5 g/L yeast extract) and incubated at 28°C for 14 days. Conidia were harvested in 40 mL sterile water and filtered through six layers of lens tissues to remove mycelial debris. The conidia were quantified with a hemocytometer [30,32].

### Analysis of trap formation and nematocidal activity

To analyze trap formation and pathogenicity of fungal strains, approximately 3 × 10^4^ conidia were inoculated in water agar plates and cultured at 28°C for 3–4 days until the hyphae covered the plate. Then, ~300 nematodes were added, and trap formation was evaluated at 12 h intervals. The captured nematodes were quantified at 24 h intervals.

### Stress tolerance test

Stress tolerance was determined as described previously [30]. The fungal strains were inoculated on TG plates supplemented with stressors such as SDS (0.01%, 0.02%, and 0.03%), NaCl (0.1, 0.2, and 0.3 M), and H_2_O_2_ (5 mM, 10 mM, and 15 mM) [30]. Relative growth inhibition (RGI) was calculated as previously described [33].

### Microscopic observation of mycelium and spores

To observe hyphal morphology, the fungal hyphae were stained with calcoﬂuor white (CFW) using the cutting cover glass method [34] and incubated on PDA medium at 28°C for 5 days. The cell wall and hyphal septum were stained with CFW (Sigma-Aldrich), as previously described [35]. The conidial nuclei were visualized by staining with 20 mg/mL 4ʹ,6-diamidino-2-phenylindole (Sigma-Aldrich) and 20 mg/mL CFW, as previously described [36]. The hyphae and conidial nuclei were observed with an inverted fluorescence microscope (Carl Zeiss, Heidenheim, Germany).

### Proteolytic activity assay

Fungal strains were incubated in PD broth at 28°C for 7 days with shaking at 180 rpm. The fermentation liquid was collected with a sterile funnel. Proteolytic activity was assayed on skim milk-plates, and proteolytic rings were observed after the plate was incubated at 37°C for 48 h [30].

### Quantification of intracellular cAMP

Intracellular cAMP level was determined as described previously [37]. Fungal strains were incubated in liquid PD medium at 28°C. Mycelia were harvested at 3, 5, and 7 d. The mycelia samples were treated with 1 M HCl for 30 min and frozen in liquid nitrogen. Intracellular cAMP was extracted and quantified using a direct cAMP ELISA kit (Enzo Life Sciences, Korea).

### Quantitative real-time PCR (RT-PCR) analysis

Total RNA was isolated from mycelial samples, and reverse transcribed into cDNA as described previously [38]. Gene-specific primers were designed with Primer3 software, and the β-tubulin gene was used as an internal reference (Table S5). RT-PCR analysis was performed as described previously [38]. The relative transcript level of each gene was calculated using the 2^−ΔΔCt^ method [39].

### Transcriptomic sequencing and analysis

The WT and Δ*AoFlbA* mutant strains were incubated on PDA plates for 7 d, then transferred into PD broth and incubated at 28°C and 180 rpm for 7 d. Mycelia were harvested by filtration and nematodes were added to induce trap formation. Then, mycelial samples were collected at 0, 6, 12, and 24 h and frozen in liquid nitrogen. Mycelial samples were sent to Shanghai Majorbio Bio-pharm Technology Co. Ltd for transcriptome sequencing. The data were analyzed using the Majorbio Cloud Platform (www.majorbio.com). Goatools software was used to perform GO enrichment analysis [40]. Significantly enriched GO functions were identified using Fisher’s exact tests and screening for an adjusted *p* value (Padjust) < 0.05. KEGG pathway enrichment analysis was performed on genes/transcripts using a custom R script [41]. Significant enrichment was identified using the same method as the GO functional enrichment analysis. After the analysis, 28 genes were selected to verify the transcriptome data using RT-PCR. The genes and primers are shown in Table S6. Sequence data were deposited in the National Microbiology Data Center (NMDC, http://nmdc.cn/resource/search) under the Accession No. NMDC40001390.

### Statistical analysis

Data are presented as the mean ± standard deviation (SD). All experiments were repeated three times. Prism 5 (GraphPad, San Diego, CA, USA) was used to generate plots and perform statistical analyses. Data were analyzed using one-way ANOVA and Tukey’s HSD. *P* < 0.05 was considered to indicate significant differences.

## Results

### Properties and conserved domains of RGSs in A. oligospora

**Figure 1.**
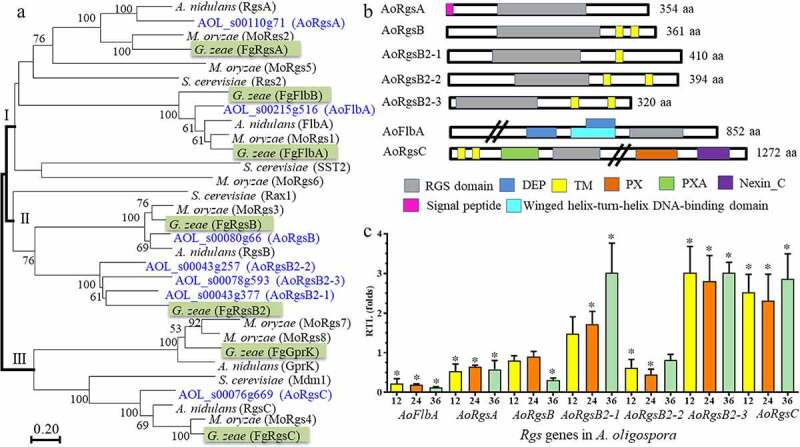
**Phylogenetic relationship among orthologous RGSs from various fungi, prediction of conserved domains, and RGS gene expression in *A. oligospora***. A. The phylogenetic tree was constructed based on amino acid sequences of RGSs from different fungi. The numbers near the nodes indicate the bootstrap values. The GenBank numbers of RGSs from different fungi used in this tree are shown in Table S7. B. The conserved domains of seven RGSs in *A. oligospora* were predicted using InterProScan. C. The relative transcript levels of seven RGS genes in *A. oligospora* during trap formation. *A. oligospora* was incubated in PD broth at 28°C for 7 days. Mycelia were collected and nematodes were added to induce trap formation. Gene expression was examined at 12, 24, and 36 h after trap induction. Error bars: standard deviation. An asterisk indicates significant differences between the transcript level of *Rgs* genes at different stages of trap development (12 h, 24 h, and 36 h) vs. the transcript at 0 h (Tukey’s HSD, *P* < 0.05)

Seven putative RGSs were identified in *A. oligospora* using orthologous RGS and RGS-like proteins from the model fungi such as *A. nidulans, M. oryzae*, and *S. cerevisiae* as queries. A phylogenetic tree was constructed using Mega v7.0 software [28], and the fungal RGSs from different fungi were grouped into three clades (Figure 1a). The model fungus *A. nidulans* contains five RGSs including RgsA, FlbA, RgsB, GprK, and RgsC. *G. zeae* contains two additional orthologs: FlbB and RgsB2. The homologous RgsA and FlbA from *A. nidulans* and other fungi were clustered in clade I. RgsB and RgsB2 from different fungi were clustered in clade II, whereas the GprK and RgsC orthologs were clustered in clade III (Figure 1a). Seven *A. oligospora* RGSs were named according to their clustering with the *A. nidulans* and *G. zeae* orthologs. Two *A. oligospora* RGSs, AOL_s00110g71 and AOL_s00215g516, were clustered in clade I and were named AoRgsA and AoFlbA, respectively. Similarly, four *A. oligospora* RGSs clustered in clade II, one of which (AOL_s00080g66) clustered with RgsB from *A. nidulans* and *G. zeae*. This RGS was named AoRgsB, whereas the other three proteins clustered with *G. zeae* RgsB2, so they were named RgsB2-1, RgsB2-2 and RgsB2-3. Finally, RGS AOL_s00076g669 in *A. oligospora* clustered with *A. nidulans* and *G. zeae* RgsC, and was named AoRgsC. *A. nidulans* and *G. zeae* GprK orthologs were not detected in *A. oligospora*.

The partial properties of RGSs in *A. oligospora* were analyzed (Table S1). Five of the RGSs ranged in molecular weight (MW) from 35.8 to 46.2 kDa, but AoFlbA and AoRgsC shared a higher MW (over 92.5 kDa). All RGSs contained a conserved RGS domain (Figure 1b). AoRgsA contained one N-terminus signal peptide (SP), but no SPs were identified in the other six RGSs. Four RGSs (AoRgsB, AoRgsB2-1, AoRgsB2-2, and AoRgsB2-3) contained one or two transmembrane (TM) domains at the C-terminus. Consistent with their higher MW, AoFlbA and AoRgsC contained more domains. For example, AoFlbA contained two DEP domains (IPR000591) and a winged helix-turn-helix DNA-binding domain (IPR036388) at the N-terminus, and AoRgsC possessed two TM domains and one PXA domain (IPR003114) at the N-terminus, and a PX (IPR001683) and nexin_C domain (IPR013937) at the C- terminus.

### Expression of seven RGS-encoding genes during trap formation and predation

To probe the possible functions of RGSs during trap formation in *A. oligospora*, their expression levels were examined by real time reverse transcription PCR (RT-PCR). *AoFlbA* was significantly downregulated compared to 0 h (4.4-, 5.2-, and 8.2-fold at 12, 24, and 36 h, respectively), especially in the later infection stage (Figure 1c). Meanwhile, *AoRgsA, AoRgsB*, and *AoRgsB2-2* were also downregulated at three time points (12, 24, and 36 h). In contrast, *AoRgsB2-3* and *AoRgsC* were significantly upregulated at 12, 24, and 36 h. Specifically, *AoRgsB2-3* increased by 2.8–3.0 fold at 12, 24, and 36 h, respectively. *AoRgsB2-1* expression gradually increased from 12 h to 36 h, with a 3.0-fold increase at 36 h (Figure 1c).

### RGSs regulate mycelial growth, development, and asexual reproduction

**Figure 2.**
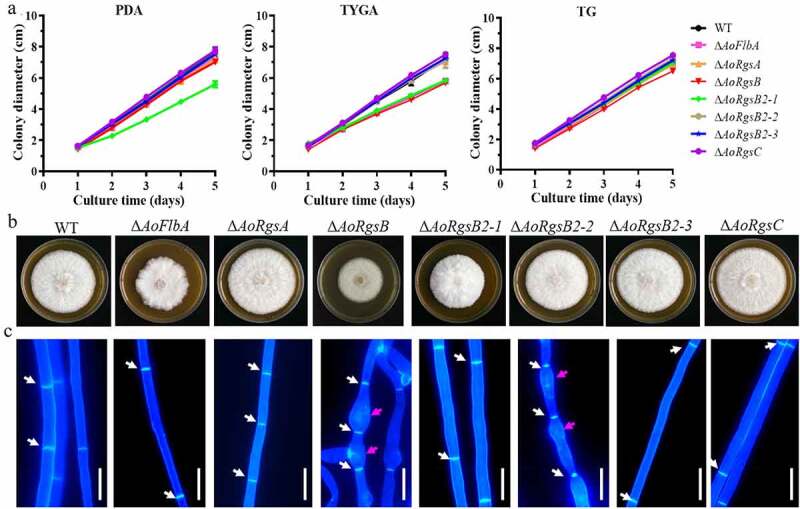
**Comparison of mycelial growth rate, colony morphology, and hyphal development between the WT and Δ*AoRgs* mutants**. A. Comparison of mycelial growth between the WT and mutants on PDA, TYGA, and TG plates. B. Colony morphology of the WT and mutants incubated on TYGA plates for 5 days at 28°C. C. Hyphal septa and cell morphology of the WT and mutants. The hyphal septa and cell morphology were observed after staining with calcoﬂuor white. White arrows: mycelial septa; red arrows: swollen cells. Bar = 10 μm

Three transformants containing the correct mutation in each *AoRgs* gene were obtained as described in Materials and methods, and identified via PCR and southern blot (Fig. S1). Since independent mutant strains of each gene showed similar phenotypic traits, a single mutant from each *AoRgs* gene was selected for subsequent study. Mycelial growth and colony morphology of the fungal strains were compared on PDA, TG, and TYGA media (Figure 2a). Two mutants (Δ*AoFlbA* and Δ*AoRgsB2-1*) showed reduced mycelial growth in all three kinds of media, and Δ*AoRgsB* mutant showed reduced mycelial growth in TYGA medium, whereas the mycelial growth of the Δ*AoRgsC* mutant was slightly increased in all three kinds of media compared to the WT strain. The Δ*AoRgsB2-1 *mutant produced dense aerial hyphae, whereas the Δ*AoRgsB* mutant produced sparse aerial hyphae (Figure 2b). The mycelial septum and cell morphology of the WT and mutant strains were observed after they were stained with CFW. The mycelial septum showed uneven distribution in the WT and mutant strains, and partial mycelia of the Δ*AoRgsB* and Δ*AoRgsB2-2* mutant strains showed abnormal swelling (Figure 2c).

**Figure 3.**
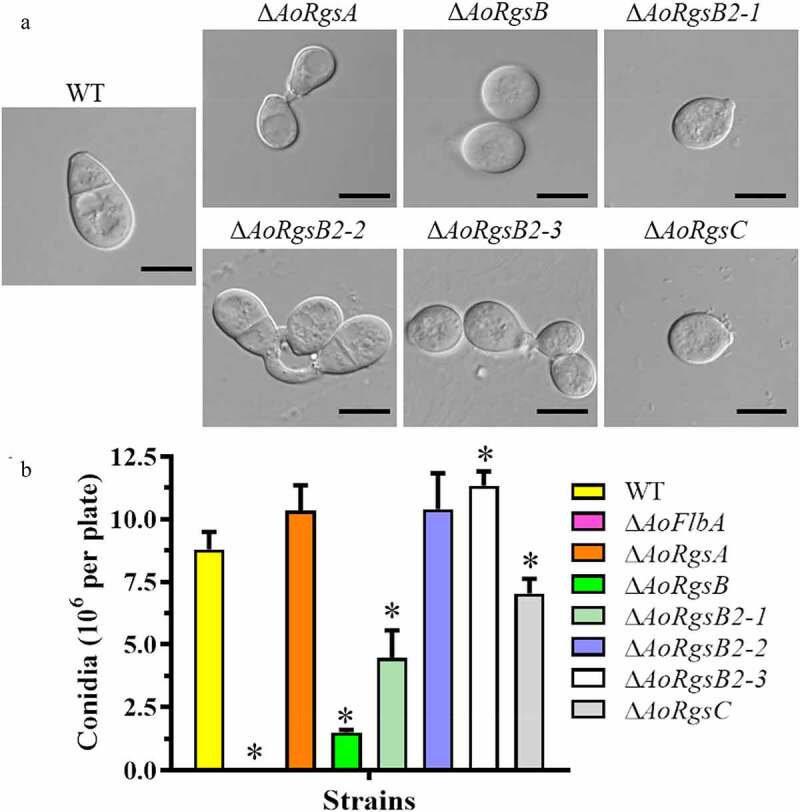
**Comparison of conidial morphology and yields between the WT and Δ*AoRgs* mutants**. A. Conidial morphology of the WT and Δ*AoRgs* mutants incubated on CMY plates at 28°C for 14 d. B. The conidia yields of the WT and Δ*AoRgs* mutants. Bar = 10 μm. Error bars: standard deviation, asterisk: significant difference between mutant and WT (Tukey’s HSD, *P* < 0.05)

To examine whether the RGSs are involved in asexual development, the fungal conidia yields were determined (Figure 3). Compared to the WT strain (8.8 × 10^6^/plate), the conidia yield was increased by 17.3, 18.2, and 28.8% in the Δ*AoRgsA*, Δ*AoRgsB2-2*, and Δ*AoRgsB2-3* mutants, respectively. The conidia yield was decreased by 83%, 49.5%, and 20% in the Δ*AoRgsB*, Δ*AoRgsB2-1* and Δ*AoRgsC* mutants, respectively (Figure 3b). Importantly, disrupting *AoFlbA* resulted in a significant defect in sporulation, and the Δ*AoFlbA* mutant lost the ability to produce conidia. In addition, partial conidia in these mutants had altered morphology. For example, spores from the Δ*AoRgsA*, Δ*AoRgsB*, Δ*AoRgsB2-1*, Δ*AoRgsB2-3*, and Δ*AoRgsC* mutants were round and lacked a transverse septum, while the WT and Δ*AoRgsB2-2* mutant conidia were obovoid or pear-shaped, with a septum near the base (Figure 3a). Moreover, fewer nuclei were observed in the deformed conidia of Δ*AoRgsA*, Δ*AoRgsB*, Δ*AoRgsB2-1*, Δ*AoRgsB2-3*, and Δ*AoRgsC* mutant strains than in those of the WT and Δ*AoRgsB2-2* mutant strains (Fig. S2).

### RGSs regulate stress resistance

**Figure 4.**
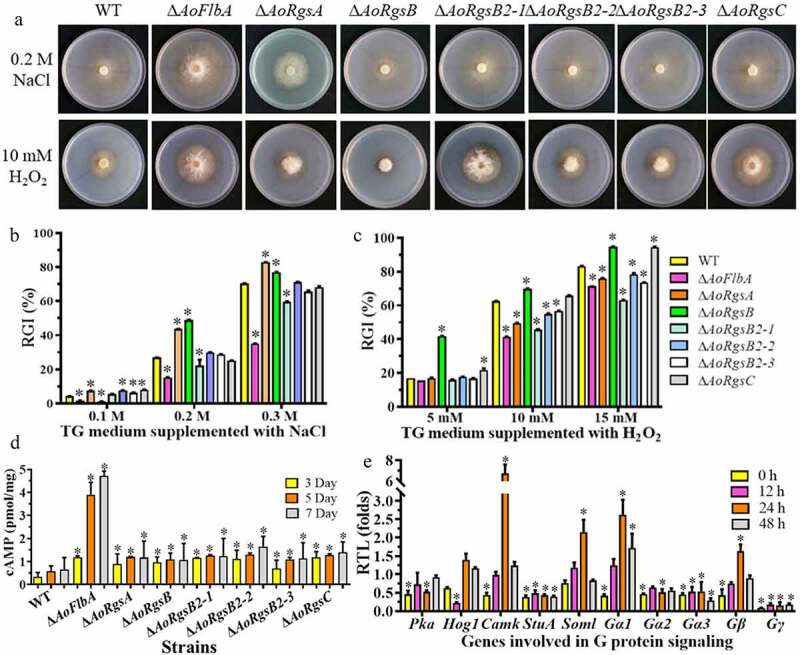
**Comparison of stress resistance, intracellular cAMP levels, and mRNA of G protein signaling components between WT and Δ*AoRgs* mutants**. A. Colony morphologies of the WT and Δ*AoRgs* mutants after incubation on TG medium containing NaCl and H_2_O_2_. B. RGI values of the fungal strains incubated on TG medium containing 0.1–0.3 M NaCl for 7 days. C. RGI values of the fungal strains incubated on TG medium containing 5–15 mM H_2_O_2_ for 7 days. D. Intracellular cAMP levels in the hyphae were measured after the WT and Δ*AoRgs* mutants were cultured in PD broth for 3, 5, and 7 d. E. The relative transcript levels of genes encoding proteins involved in G protein signaling in the WT and Δ*AoFlbA* mutant. The WT and Δ*AoFlbA* mutant were incubated in PD broth at 28°C for 7 days. Mycelia were collected and induced by nematodes for 12, 24, 36, and 48 h. Error bars: standard deviation, asterisk: significant difference between the Δ*AoRgs* mutants and the WT strain (Tukey’s HSD, *P* < 0.05)

The fungal strains were inoculated on TG plates supplemented with chemical stressors [H_2_O_2_, sodium dodecyl sulfate (SDS), and NaCl]. These chemicals significantly inhibited the growth of WT and Δ*AoRgs* mutants, with varying degrees of RGI values (Figure 4). As shown in Figure 4a, SDS significantly inhibited mycelium growth in both WT and mutants, but the SDS RGI values were not significantly different except in Δ*AoRgsA* at 0.02% SDS and Δ*AoRgsB2-1* at 0.02–0.03% SDS (Fig. S3). In contrast, the RGI values of Δ*AoRgs* mutants grown with NaCl were significantly different from the WT strain (Figure 4b). A low NaCl concentration (0.1 M) reduced the RGI values of Δ*AoRgsB* and Δ*AoFlbA* mutants, and increased the RGI values in other mutants. Further, the RGI values of Δ*AoRgsA* and Δ*AoRgsB* mutants were significantly increased in medium containing 0.2–0.3 M NaCl, and the Δ*AoFlbA* and Δ*AoRgsB2-1* RGI values were significantly decreased. Similarly, the RGI value of Δ*AoRgs* mutants was significantly different from that of WT on H_2_O_2_ media. The RGI values of Δ*AoRgsB* and Δ*AoRgsC* mutants was increased at 5–15 mM H_2_O_2_, while the other mutants showed enhanced antioxidant capacity at 10–15 mM H_2_O_2_. The RGI values of Δ*AoFlbA* (71.34%) and Δ*AoRgsB2-1* (62.88%) mutants were significantly decreased compared to the WT strain (83.3%) on TG plates supplemented with 15 mM H_2_O_2_ (Figure 4c).

### RGSs regulate trap formation, proteolytic activity, and nematocidal activity

**Figure 5.**
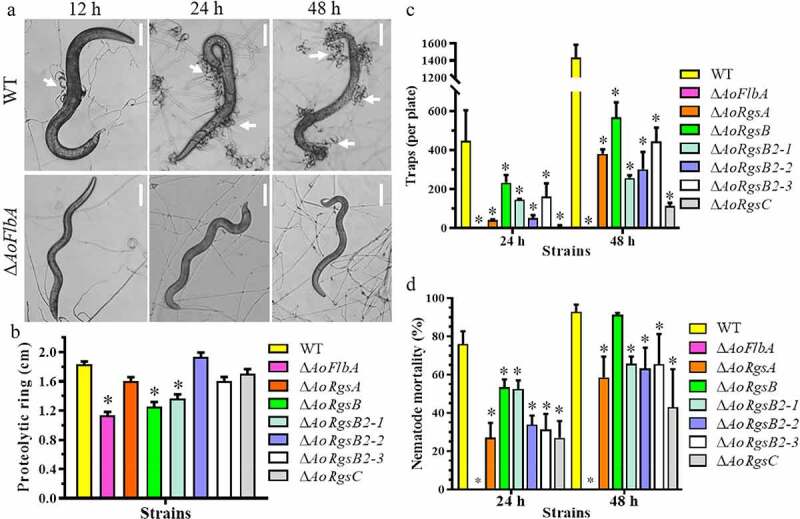
**Comparison of trap formation, nematocidal activity, and proteolytic activity between the WT and Δ*AoRgs* mutants**. A. Nematode-induced trap formation in the WT and Δ*AoRgs* mutants. The WT and Δ*AoFlbA* mutant were used as examples. Bar = 10 μm. White arrows indicate the traps. B. Comparison of extracellular proteolytic activity between the WT and Δ*AoRgs* mutants. The transparent zone produced due to the degradation of protein substrate in the plate. C. The number of traps produced by the WT and Δ*AoRgs* mutants induced by nematodes at 24 h and 48 h. D. The percentage of nematodes captured by the WT and Δ*AoRgs* mutants at 24 h and 48 h. Error bars: standard deviation, asterisks: significant differences between the Δ*AoRgs* mutants and the WT strain (Tukey’s HSD, *P* < 0.05)

To determine whether RGSs are involved in the regulation of trap formation in *A. oligospora*, WT and Δ*AoRgs* mutants trap formation was compared. The WT strain produced immature traps containing one or two hyphal loops, and few nematodes were captured after 12 h. Mature traps containing multiple hyphal loops were observed and more nematodes were captured at 24 h. Most of the nematodes were captured at 36 h and digested by 48 h (Figure 5a, Figure 5d). The Δ*AoRgs* mutants showed significant defects in trap formation. The Δ*AoRgsC* mutant only produced 0.08-fold as many traps as the WT strain, and no trap formation was observed in the Δ*AoFlbA* mutant after induction for 48 h (Figure 5a, Figure 5c). Additionally, the nematocidal activity of the Δ*AoRgs* mutants was decreased by different degrees at different time points. The WT strain captured 24.3%, 76.0%, 85.5% and 92.9% of nematodes at 12, 24, 36, and 48 h, respectively. Relative to the WT, the Δ*AoRgsC* mutant captured 9.6%, 27.0%, 35.8%, and 43.1% of nematodes at the same four time points, respectively. Further, the Δ*AoFlbA* mutant was unable to capture and degrade nematodes (Figure 5d).

Subtilisin-like proteases of pathogenic fungi are a large family of extracellular cuticle-degrading enzymes that contribute to fungal invasion into their hosts through cuticle infection [42]. To determine whether Δ*AoRgs* mutants are involved in protease production, proteolytic activity was analyzed using skim milk plates [30]. Compared to the WT strain, most of the Δ*AoRgs* mutants showed reduced proteolytic activity, especially the Δ*AoRgsB*, Δ*AoRgsB2-1*, and Δ*AoFlbA* mutants. However, the Δ*AoRgsB2-2* mutant showed a slight increase in proteolytic activity (Figure 5b).

### RGSs regulate intracellular cAMP levels and the transcripts of G protein signaling genes

To determine whether RGSs are involved in the regulation of cAMP accumulation, intracellular cAMP levels in the WT and Δ*AoRgs* mutant hyphae were measured after culture in PD broth for 3, 5, and 7 d. Our results indicated that cAMP levels were increased in the Δ*AoRgs* mutants compared to the WT, and that the cAMP level in the Δ*AoFlbA* mutant was significantly higher than in the other mutants (Figure 4d). Indeed, the Δ*AoFlbA* mutant showed 3.6-, 3.3-, and 7.2-fold higher cAMP levels compared to the WT strain at 3, 5, and 7 d, respectively.

Moreover, the expression of several genes associated with the G protein signaling pathway in the WT and Δ*AoFlbA* mutants was compared during trap formation and predation stages. Compared to the WT strain, the genes encoding PKA, Gα2, Gα3, Gγ, and StuA were downregulated in the Δ*AoFlbA* mutants during trap formation and predation stages. In particular, *StuA* and *Gγ* expression were significantly downregulated (Figure 4e). *Gγ* expression was decreased by 11.6, 5.6, 6.7, and 5.5-fold at 0, 12, 24, and 36 h, respectively. Interestingly, *Gα1, Gβ*, and *Soml* showed a similar pattern. The expression of these genes gradually increased at 0, 12, and 24 h, then decreased from 24–36 h.

### Transcriptomic profile analysis of the AoFlbA gene by RNA-sequencing

Based on the phenotypic analysis of Δ*AoRgs* mutants, the Δ*AoFlbA* mutant was chosen for further study by RNA-sequencing. WT and mutant mycelial samples were incubated with nematodes for 0, 6, 12, and 24 h. Then, cDNA libraries were constructed and sequenced. The number of raw and clean reads are shown in Table S3. After removing adapter and low-quality sequences, an average of 45.29 million reads were obtained per sample. The percentage of Phred-like quality scores at the Q30 level (an error probability of 1%) ranged from 93.4% to 94.5%, and the GC content was estimated to be 47.2% to 48.4% (Table S3). Principal component analysis showed close proximity and some overlap between samples at the same time point, indicating high similarity and good reproducibility of the three replicates (Fig. S4). To verify the transcriptomic data, 28 genes were examined using RT-PCR. The results showed that all the selected genes had the same expression pattern (Fig. S5).

**Figure 6.**
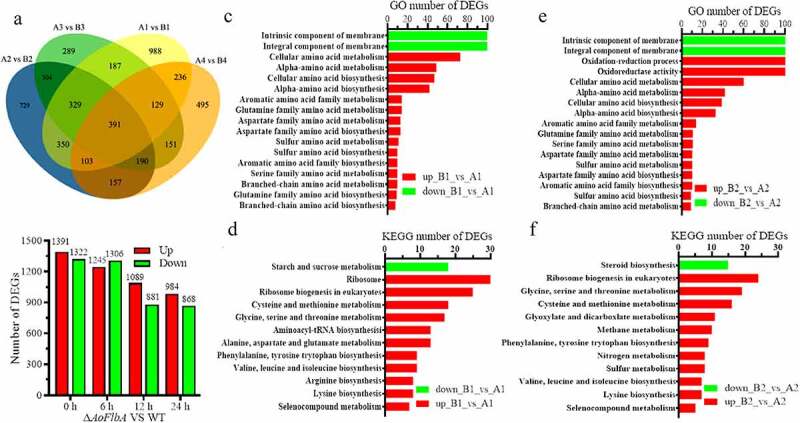
**Comparison of differentially expressed genes (DEGs) between the WT and Δ*AoFlbA* mutants**. A. Venn diagram of DEGs in the WT (a) and Δ*AoFlbA* (b) mutant during vegetative growth and trap formation. Each color represents a time point. Yellow represents 0 h, blue is 6 h, green is 12 h, and orange is 24 h. A1 (B1), A2 (B2), A3 (B3), and A4 (B4) indicate mycelial samples at 0, 12, 36, and 48 h. B. The number of upregulated and downregulated DEGs in the Δ*AoFlbA* mutant versus WT at different time points. C. GO enrichment analysis of DEGs in the Δ*AoFlbA* mutant versus the WT strain at 0 h. D. KEGG enrichment analysis of DEGs in the Δ*AoFlbA* mutant versus the WT strain at 0 h. E. GO enrichment analysis of DEGs in the Δ*AoFlbA* mutant versus the WT strain at 6 h. F. KEGG enrichment analysis of DEGs in Δ*AoFlbA* mutant versus WT strain at 6 h. Red indicates upregulated genes, and green represents downregulated genes

Differentially expressed genes (DEGs) were identified by comparing the TPM (transcripts per kilobase million) values of each gene between the WT and Δ*AoFlbA* mutant strains. Compared to the WT strain, there were 2713, 2553, 1970, and 1852 DEGs in the mutant at 0, 6, 12, and 24 h, respectively (Figure 6a, Figure 6b). Then, the significantly enriched pathways were examined at each time point by performing gene ontology (GO) analysis. The most significant changes in gene expression occurred at 0 and 6 h (Figure 6c, Figure 6e), and these time points had the highest number of significantly enriched pathways. Compared to the WT strain, there was a significant upregulation in numerous genes related to amino acid metabolic and biosynthetic processes in *∆AoFlbA* mutant at 0 h, such as alpha-amino acid, glutamine family amino acid, aspartate family amino acid, sulfur amino acid, serine family amino acid, and branched-chain amino acid (Figure 6c). In addition to amino acid metabolic and biosynthetic processes, oxidation-reduction process and oxidoreductase activity were significantly upregulated at 6 h (Figure 6e). A similar pattern of DEGs enrichment was observed at 12 h. In addition to the processes upregulated at 6 h, catalytic activity, cofactor binding, organic acid metabolic process, and transmembrane transport were significantly upregulated at 12 h (Fig. S6A). However, only oxidation-reduction process and oxidoreductase activity were significantly upregulated at 24 h (Fig. S6B). Compared with the upregulated processes, the downregulated DEGs were mainly enriched in the integral component of membrane and intrinsic component of membrane at 0, 6, and 12 h (Figure 6c, Figure 6e, S5A). These results indicate that AoFlbA may be involved in regulating genes related to cell membrane synthesis and integrity in *A. oligospora*.

The significantly up- and downregulated pathways at each time point were determined by KEGG enrichment. Most of the pathways that were significantly upregulated during vegetative growth and trap formation were ribosome biogenesis, and diverse amino acid metabolism and biosynthesis, such as cysteine and methionine metabolism, phenylalanine, tyrosine, and tryptophan biosynthesis at 0 and 6 h (Figure 6d, Figure 6f). Peroxisome and fatty acid degradation were also enriched at 12 h (Fig. S7A), and only two pathways (tyrosine metabolism and glycine, serine and threonine metabolism) were enriched at 24 h (Fig. S7B). Similar to GO analysis, few DEGs were enriched in downregulated pathways, including steroid biosynthesis and starch and sucrose metabolism.

### The Gas1 gene family expands in A. oligospora and is differentially expressed during trap formation

**Figure 7.**
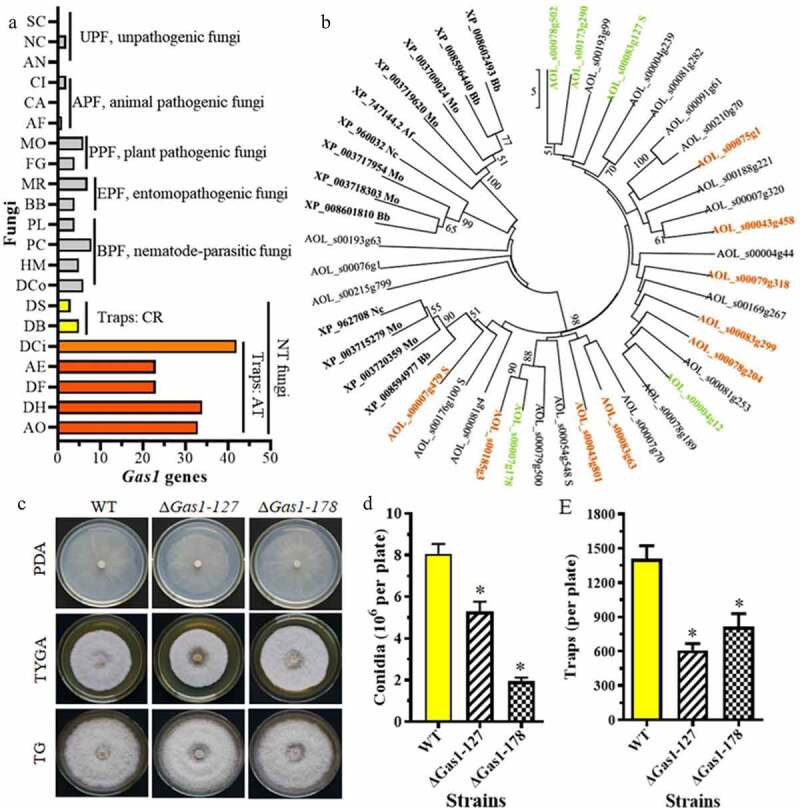
**Comparison of *Gas1* genes in diverse fungi and differentially expressed *Gas1* genes in *A. oligospora***. A. Comparison of the number of *Gas1* genes in diverse fungi. *Gas1* genes in diverse fungi were retrieved from GenBank. *A. oligospora Gas1* genes were used as the queries. B. Cluster and transcript analyses of *Gas1* genes in *A. oligospora*. The genes marked in color have altered mRNA expression. Orange is upregulated and green is downregulated. The abbreviations of fungal strains are shown in Table S8. **C**. Colonies of the WT and Δ*Gas1* mutants incubated on PDA, TG, and TYGA plates at 28°C for 5 days. D. Conidia yields of the WT and Δ*Gas1* mutants incubated on CMY plates at 28°C for 14 days. E. The number of traps produced by the WT and Δ*Gas1* mutants induced by nematodes for 48 h

Gas1 is an ortholog of Egh16/Egh16H family members, which are highly expressed in the infection stage and are involved in pathogenesis [43,44]. There are few *Gas1* homologous genes in unpathogenic fungi (UPF) and animal pathogenic fungi (APF), and none are found in *S. cerevisiae, A. nidulans*, or *Coccidioides immitis*. More *Gas1* genes are observed in plant pathogenic fungi (FFP) and entomopathogenic fungi (EPF). For example, there are 4 and 6 *Gas1* genes in the FFP *M. oryzae* and *G. zeae*, respectively. Similarly, 4 and 7 *Gas1* genes were identified in the EPF *Beauveria bassiana* and *Metarhizium robertsii*, respectively. A similar number of *Gas1* genes (from 4 to 8) were found in nematode-parasitic fungi (NPF). For example, 4 and 8 *Gas1* genes were found in the NPF *Pochonia chlamydosporia* and *Purpureocillium lilacinum*, respectively. The *Gas1* genes in NT fungi that produce adhesive traps (e.g., adhesive networks, adhesive knobs, and adhesive columns) were significantly expanded (23–42 genes), and 33, 34, and 42 *Gas1* genes were found in *A. oligospora, Dactylellina haptotyla*, and *D. cionopaga*, respectively. The number of *Gas1* genes in NT fungi that produce constricting rings (CR) was no different from that in NPF. There were only 3 and 5 *Gas1* genes identified in the CR-producing fungi *Drechslerella brochopaga* and *Drechslerella stenobrocha*, respectively (Figure 7a).

Transcriptomic analysis showed that the expression of *Gas1* family genes was significantly altered during trap formation. Nine genes (e.g., *AOL_s00043g458, AOL_s00083g299, AOL_s00007g479*, and *AOL_s00185g3*) were upregulated during trap formation, and five genes (e.g., *AOL_s00078g502, AOL_s00083g127, AOL_s00007g178*, and *AOL_s00173g290*) were downregulated (Figure 7b; Table S4). To verify the role of *Gas1* genes, we knocked out two genes encoding Gas1 proteins in *A. oligospora: AOL_s00083g127* (*Gas1-127*) and *AOL_s00007g178* (*Gas1-178*). Deleting *Gas1-127* and *Gas1-178* significantly reduced conidiation and trap formation compared to the WT strain (Figure 7c, Figure 7d). The conidia yield of *Gas1-127* and *Gas1-178* deletion mutants was decreased by 33.2% and 76.1%, respectively, compared to the WT strain. Similarly, trap formation in *Gas1-127* and *Gas1-178* mutants was decreased by 57.6% and 43.5%, respectively (Figure 7e).

## Discussion

G proteins have universal roles as signaling proteins in eukaryotes and play an indispensable role in regulating fungal growth, asexual reproduction, and pathogenicity [1,5,45]. RGSs act as GTPase-activating proteins to negatively control G protein signaling [1]. Recently, an increasing number of RGSs have been identified in diverse fungi. For example, four RGS and RGS-like proteins were identified in *S. cerevisiae* [2], five RGSs in *A. nidulans* [16], and eight RGSs in *M. oryzae* [17]. These studies suggest that RGSs play important roles in mycelial growth, sexual and asexual reproduction, secondary metabolite formation, and pathogenicity [14,17,46]. In this study, seven putative *A. oligospora* RGSs were functionally characterized. Our analysis showed that the seven RGSs play pleiotropic roles in vegetative growth, asexual development, stress tolerance, trap formation, and pathogenicity.

RGSs contain highly conserved RGS domains in diverse fungi, but mutations in these genes often result in different phenotypic changes in fungi. AoFlbA in *A. oligospora* is highly homologous to MoRgs1 in *M. oryzae* and *FlbA* in *A. nidulans. AoFlbA* was significantly downregulated in the WT strain during trap formation and predation stages, and deleting *AoFlbA* resulted in significant cAMP accumulation in hyphae compared to the WT strain. Meanwhile, *AoFlbA* deletion caused a defect in mycelial growth and enhanced resistance to H_2_O_2_ and NaCl. Importantly, the Δ*AoFlbA* mutant lost the ability to produce spores and traps, which is consistent with studies on the positive regulation of asexual and pathogenic development by *FlbA* in *A. nidulans* and *MoRgs1* in *M. oryzae* [17,47]. MoRgs1 functions as a prominent RGS in *M. oryzae*, where it regulates surface hydrophobicity, conidiation, and mating, and is important for germ tube growth and appressorium formation [17]. Similarly, the spores produced by the *FlbA* deletion mutant decreased by 59% in *G. zeae*. These mutants could only colonize inoculated wheat, but could not spread to the surrounding wheat [14]. These results indicate that *FlbA* orthologs are prominent RGSs, which may have a negative effect on Gα signaling and regulate hyphal growth, asexual reproduction, and virulence via the cAMP/PKA signal transduction pathway in fungi.

RgsA is a conserved regulator and is also downregulated during the trap formation and predation stages. Deleting *AoRgsA* in *A. oligospora* increases the conidia yield and reduces trap formation, but resulted in no significant difference in hyphal growth compared to the WT strain. Interestingly, knockout of *RgsA* in *G. zeae* and *A. nidulans* results in a significant reduction in the growth rate of the mutant and an increase in spore production, respectively [14,15]. However, deleting *RgsA* causes an increase in radial growth and sporulation in *A. fumigatus* [48]. Meanwhile, the Δ*RgsA* mutant produces an increased amount of deoxynivalenol and zearalenone in *G. zeae* [14]. Therefore, RgsA orthologs are involved in the regulation of hyphal growth, conidiation, virulence, and secondary metabolism, whereas its roles vary in different fungi.

RgsB is also conserved in diverse fungi, and *AoRgsB* is downregulated during the trap formation and predation stages. Deleting *AoRgsB* in *A. oligospora* caused a reduction in hyphal growth, conidial yield, trap formation, and nematocidal activity. Meanwhile, the Δ*AoRgsB* mutant was more sensitive to NaCl and H_2_O_2_, and partial hyphal cells in the mutant became swollen. Similarly, deleting *Rgs3* (orthologous to *RgsB*) in *M. oryzae* alters asexual reproduction, growth of germinal tubes, and formation of appressorium [17]. When the homologous gene *RgsB* in *G. zeae* is deleted, vegetative growth was reduced, conidia production was increased, conidia morphology was changed, and virulence was weakened [14]. Thus, RgsB regulates sexual and asexual reproduction, conidial morphology, mycelial growth, and pathogenicity in fungi.

RgsC was identified in various fungi, and shares more conserved domains than other members. *AoRgsC* was significantly upregulated during trap formation. Deleting *AoRgsC* caused a defect in conidia yield, conidia morphology, trap formation, and nematocidal activity in *A. oligospora*. Meanwhile, the Δ*AoRgsC* mutant showed increased sensitivity to H_2_O_2_. RgsC orthologs regulate sexual and asexual reproduction in *M. oryzae* and *G. zeae*. Conidiation in the mutant Δ*MoRgs4* (the ortholog of *RgsC*) was reduced to 0.038-fold [17]. The Δ*FgRgsC* deletion mutants exhibited a specific defect in conidia morphology and sexual development, which produced normal perithecia, but the number of ascospores discharged from the perithecia was dramatically reduced [14]. Therefore, RgsC plays a role in conidiation, spore morphology, sexual development, and virulence in different fungal species.

Unlike other RGSs, three RGSs (AoRgsB2-1, AoRgsB2-2 and AoRgsB2-3) in *A. oligospora* were clustered with *G. zeae* RgsB2. Deletion of *AoRgsB2-1* reduced conidiation and trap formation, while deletion of *AoRgsB2-2* and *AoRgsB2-3* increased conidiation and reduced trap formation. However, deletion of *FgRgsB2* did not cause significant differences in conidiation and virulence compared to WT *G. zeae* [14]. Therefore, RgsB2 is expanded in *A. oligospora*, and plays a role in conidiation, spore morphology, trap formation, and nematocidal activity.

cAMP is a second messenger of G protein signaling. Previous studies have shown that RGSs play an important role in regulating the intracellular cAMP level in *M. oryzae* [17,37]. Compared to the WT strain, cAMP levels were signiﬁcantly higher in the Δ*MoRgs1* mutant [17,37]. Although abnormal cAMP accumulation in the mutants results in significantly accelerated appressoria formation, pathogenicity is remarkably lower since the appressoria do not develop into normal hyphae [17,49]. Consistently, our results showed that cAMP levels in the hyphae of the seven Δ*AoRgs* mutants were significantly increased, particularly in the Δ*AoFlbA* mutant. However, contrary to other fungi, *AoRgs* plays a positive regulatory role in infection structure formation, and deletion of *AoRgs* resulted in significantly reduced trap formation, in line with the observed pathogenic defects. These results suggest that RGSs have important roles in regulating intracellular cAMP levels and pathogenicity in fungi.

To probe the influence of RGSs on the expression of genes associated with G protein signaling, we compared the mRNA levels of genes encoding G protein subunits (α, β, and γ), Pka, Soml, and StuA in the Δ*AoFlbA* mutant. *Gα2, Gα3, Gγ, Pka*, and *StuA* expression was downregulated, and *Gα1, Gβ*, and *Som1* mRNA showed a similar trend, but their expression was increased during early trap formation and decreased at the predation stage. In *M. oryzae*, the G protein and cAMP/PKA signaling pathways are essential for the regulation of appressorium development and pathogenicity [17,50]. Similarly, Gγ and StuA are required for trap formation in *A. oligospora* [20,31]. These results indicate that AoFlbA regulates the expression of G protein subunits and downstream effectors, thus regulating downstream cellular processes.

In recent years, transcriptomic analysis has been routinely used to study the interaction between fungi and their hosts [51]. According to our phenotype analysis, RGSs play a pleiotropic role in fungal growth, development, and virulence. Importantly, homologous RGSs vary in different fungi. Among them, FlbA is a prominent RGS that plays a vital role in various fungi. Deleting *AoFlbA* abolished the ability of *A. oligospora* to produce spores and traps. To understand the effect of AoFlbA on mycelial growth and trap formation in *A. oligospora*, we compared the transcriptomic profiles of WT and Δ*AoFlbA* mutant strains. GO and KEGG enrichment analyses revealed significant gene enrichment in various biological processes, including amino acid metabolism and biosynthesis during vegetative growth and trap formation. The downregulated DEGs were enriched in carbohydrate metabolism and membrane-associated biological processes. In *M. oryzae*, proteomics analysis found that the expression of some proteins related to amino acid metabolism in Δ*MoRgs* mutants have undergone significant changes, which varied in different Δ*MoRgs* mutant strains. In addition, RGSs have regulatory effects on nutrition and exogenous nitrogen sources, and the addition of exogenous amino acids during the culture process can restore the growth defects present in Δ*MoRgs* mutants [52]. Our transcriptomic analysis also showed that AoFlbA participates in nitrogen metabolism in *A. oligospora*, and numerous biological processes related to amino acid metabolism and biosynthesis are significantly enriched during vegetative growth and trap formation. These results indicate that AoFlbA may regulate amino acid metabolism to affect vegetative growth, conidiation, and lifestyle switching in *A. oligospora*.

Moreover, the DEGs were mainly enriched in various amino acid metabolism and biosynthetic pathways during the vegetative growth stage. Oxidation-reduction processes, oxidoreductase activity, and nitrogen metabolism were enriched during early trap formation (induced by nematodes for 6–12 h), and peroxisome, fatty acid degradation, and terpenoid backbone biosynthesis were enriched after 12 h. Peroxisomes involve fatty acid *β*-oxidation, the glyoxylate cycle, hydrogen peroxide detoxification, and secondary metabolite biosynthesis [53]. Several *Pex* genes involved in peroxisome biogenesis have been identified in FFP, such as *M. oryzae* [54,55] and *F. graminearum* [56], and play a crucial role in pathogenicity. *AoStuA* deletion causes a defect in peroxisome biogenesis in *A. oligospora*, and the mutant is unable to produce traps [31]. Moreover, the Δ*AoFlbA* mutant showed increased resistance to H_2_O_2_ compared to the WT strain. Similarly, the DEGs were significantly enriched in intrinsic and integral membrane components during vegetative growth and trap formation, which coincides with increased NaCl resistance in the Δ*AoFlbA* mutant. Thus, AoFlbA regulates diverse biological processes and affects trap formation and pathogenicity in *A. oligospora*.

Gas1 is an Egh16 ortholog which was characterized in partial filamentous fungi, such as *Metarhizium acridum* and *M. oryzae*. In *M. acridum*, Gas1 is only expressed during the appressorium formation stage [43], its expression is significantly higher at the same stage in *M. orzyae* [44]. Further studies showed that *Gas1* deletion resulted in reduced virulence [43,44]. Interestingly, *Gas1* genes are significantly expanded in several NT fungi that produce adhesive traps, such as *A. oligospora, D. haptotyla*, and *D. cionopaga*. The number of Gas1 homologous genes in *A. oligospora, D. haptotyla*, and *D. cionopaga* (over 30 *Gas1* genes) is much higher than in other fungi, even compared to the NT fungi *D. stenobrocha* (3 *Gas1* genes) and *D. brochopaga* (5 *Gas1* genes). Both *D. stenobrocha* and *D. brochopaga* produce CR for nematode predation, thereby capturing nematodes through mechanical force, whereas adhesive trap-producing fungi capture nematodes using adhesive materials on the trap surface [21]. A previous study showed that a *Gas1* gene homolog, *mas3*, is specifically expressed in traps (adhesive knobs) in *Monacrosporium haptotylum* (syn. *D. haptotyla*) [57]. Further analysis showed that many *Gas1* genes were differentially expressed during trap formation. We verified the role of *Gas1* genes in *A. oligospora* by disrupting two *Gas1* genes (*Gas1-127* and *Gas1-178*). Both mutants showed significantly reduced conidiation, trap formation, and nematocidal activity. Therefore, *Gas1* family genes play a vital role in the development of infection structures in *A. oligospora* and other pathogenic fungi, thus affecting their virulence.

In this study, we identified seven putative *A. oligospora* RGSs, which are named according to orthologous RGSs from model fungi, including *S. cerevisiae* and *A. nidulans*. Phenotypic analysis showed that the seven RGSs play a pleiotropic role in hyphal growth, conidiation, stress resistance, trap formation, and virulence. Among those, AoFlbA is indispensable for conidiation and trap formation. Meanwhile, transcriptomic analysis comparing the WT and Δ*AoFlbA* mutant identified DEGs that are significantly enriched in amino acid metabolism and biosynthesis during vegetative growth and trap formation, suggesting that AoFlbA may regulate amino acid metabolism and affect trap formation in *A. oligospora*. Moreover, *Gas1* family genes are differentially expressed during trap formation. Deleting *Gas1* genes reduces trap formation and pathogenicity, suggesting that *Gas1* family genes may be involved in trap formation. Our results provide a novel insight into the role of RGSs in fungal growth, development, and differentiation. Further, our results also provide a basis for elucidating the lifestyle transition mechanisms of these fungal species, which could lead to the application of NT fungi as biocontrol agents against pathogenic nematodes.

## Supplementary Material

Supplemental Material
